# Astrocyte regulation of sleep circuits: experimental and modeling perspectives

**DOI:** 10.3389/fncom.2012.00065

**Published:** 2012-08-28

**Authors:** Tommaso Fellin, Jeffery M. Ellenbogen, Maurizio De Pittà, Eshel Ben-Jacob, Michael M. Halassa

**Affiliations:** ^1^Department of Neuroscience and Brain Technologies, Istituto Italiano di TecnologiaGenova, Italy; ^2^Sleep Division, Department of Neurology, Massachusetts General HospitalBoston, MA, USA; ^3^Division of Sleep Medicine, Harvard Medical SchoolBoston, MA, USA; ^4^School of Physics and Astronomy, Tel Aviv UniversityRamat Aviv, Israel; ^5^Center for Theoretical Biological Physics, University of California San DiegoLa Jolla, CA, USA; ^6^Department of Psychiatry, Massachusetts General HospitalBoston, MA, USA; ^7^Department of Brain and Cognitive Science, Massachusetts Institute of TechnologyCambridge, MA, USA

**Keywords:** glia, astrocytes, sleep, ATP, adenosine, neuronal networks, slow oscillations, computational models

## Abstract

Integrated within neural circuits, astrocytes have recently been shown to modulate brain rhythms thought to mediate sleep function. Experimental evidence suggests that local impact of astrocytes on single synapses translates into global modulation of neuronal networks and behavior. We discuss these findings in the context of current conceptual models of sleep generation and function, each of which have historically focused on neural mechanisms. We highlight the implications and the challenges introduced by these results from a conceptual and computational perspective. We further provide modeling directions on how these data might extend our knowledge of astrocytic properties and sleep function. Given our evolving understanding of how local cellular activities during sleep lead to functional outcomes for the brain, further mechanistic and theoretical understanding of astrocytic contribution to these dynamics will undoubtedly be of great basic and translational benefit.

## Introduction

**Figure 1:**
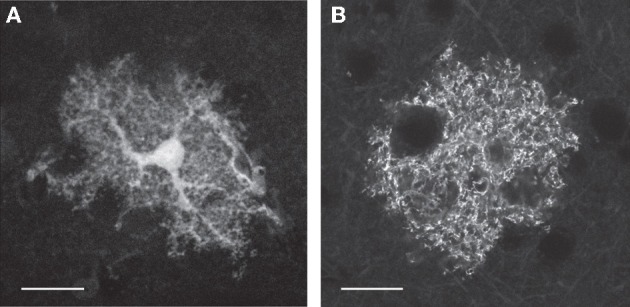
**Astrocytes are characterized by complex morphology. (A,B)** Single-plane fluorescence images of an astrocyte expressing soluble GFP **(A)** or a membrane-bound YFP **(B)**. In **A** the cell body and the principal cellular processes are clearly visible. When the fluorophore is bound to the membrane **(B)**, small distal processes are more easily identified because of the increased surface-to-volume ratio of these thin cellular compartments. Scale bar 20 μm. Courtesy of A. M. De Stasi and T. Fellin.

Astrocytes are characterized by a highly ramified structure of cellular processes that occupy non-overlapping domains (see Figure 1). Each astrocyte can contact a few neuronal cell bodies, hundreds of dendrites, and tens of thousands of synapses (Bushong et al., 2002; Halassa et al., 2007). Moreover, astrocytes can be coupled by gap junctions (Cotrina et al., 1998; Giaume et al., 2010; Verkhratsky, 2011) to form a cellular network called “astrocytic syncytium.” This elaborate morphology underlies the complexity of astrocyte function in the brain. Indeed, the impact of astrocytes on synaptic physiology is multifaceted, ranging from structural support to the regulation of the composition of the extracellular space and neuromodulation (for reviews see Haydon, 2001; Fellin and Carmignoto, 2004; Allen and Barres, 2005; Volterra and Meldolesi, 2005; Barres, 2008; Fellin, 2009).

While astrocytes lack active membrane properties (Zhou et al., 2009), their passive properties are crucial to regulating extracellular potassium (Higashi et al., 2001; Kucheryavykh et al., 2007). Astrocytes also clear extracellular excitatory and inhibitory neurotransmitters via specific transporters, a process that ensures high fidelity synaptic transmission (Kimelberg et al., 1986; Dabir et al., 2006).

**Figure 2:**
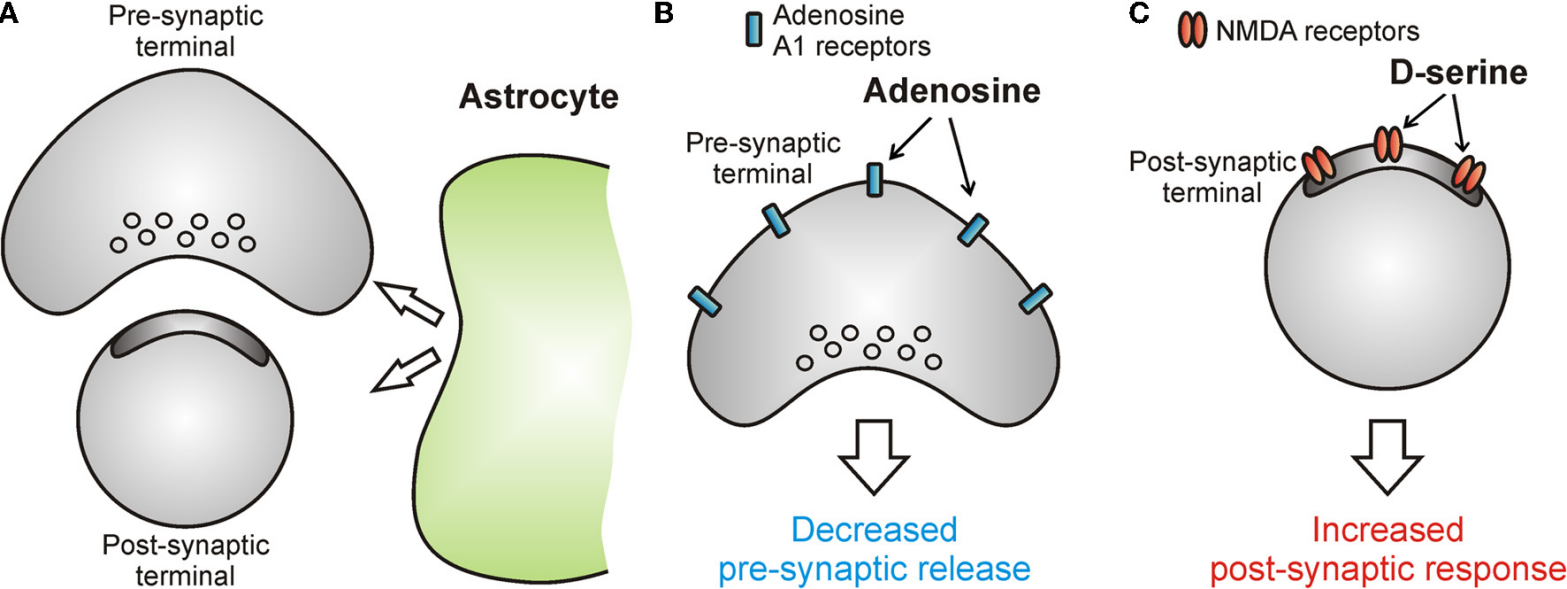
**Astrocytes release chemical transmitters to modulate information transfer at the synapse. (A–C)** Schematic drawings showing the effect of astrocytic neuromodulation on synaptic function. Astrocytes release ATP which, after rapid extracellular degradation to adenosine, activates adenosine A_1_ receptors located at pre-synaptic sites and leads to a decrease in the release of neurotransmitter **(B)**. Astrocytes can also release D-serine which potentiates the current flowing through post-synaptic NMDA receptors thus leading to increased post-synaptic responses **(C)**.

One of the most exciting discoveries in modern neuroscience has been the demonstration that astrocytes release chemical transmitters (Figure 2) among which D-serine and ATP are two of the most studied. D-serine increases the current flowing through NMDA receptors by acting as a co-agonist and providing an important regulatory feedback that controls synaptic transmission and plasticity (Panatier et al., 2006) (Figure 2C). In contrast, ATP binds to purinergic receptors (Gordon et al., 2005), or activates adenosine A_1_ and A_2_ receptors (through its metabolite, Adenosine) to modulate neuronal excitability and information transfer at the synapse (Pascual et al., 2005; Panatier et al., 2011) (Figure 2B). Two studies extended these initial findings, demonstrating that astrocytes provide powerful modulation of sleep dynamics and behavior (Fellin et al., 2009; Halassa et al., 2009). Taken together, these observations strongly support the notion that local astrocytic modulation at the synaptic level translates into an active control of network activities, such that higher brain functions are served by the integrated circuits of neurons and glia (Halassa and Haydon, 2010).

This review aims to frame such recent findings on the active role of astrocytes in sleep and behavior in light of our evolving understanding of sleep phenomenology and function, focusing on sleep's expression in forebrain circuits. Moreover, we pay particular attention to the computational implications of such findings in the attempt to build a theoretical framework for future experiments. This endeavor will allow us and others to conceptualize what is currently known about astrocytic modulation of neuronal network function and test new computationally inspired hypotheses. We hope that a community-wide effort of similar motivation will bridge the gap between experiments and models, in order to promote better understanding of the function of integrated circuit function in the brain.

## Sleep: background and function

Although details can differ among animals, a number of common characteristics have been used to define sleep as a universal behavior (Cirelli and Tononi, 2008). Among them are changes in posture, reduced responsiveness to environmental stimuli, and changes in global brain activity (Bjorness and Greene, 2009). Such characteristics are based on mammalian physiology, which traditionally classifies sleep into categories of rapid eye movement (REM) and non-rapid eye movement (NREM) stages using electroencephalographic (EEG) and electromyographic (EMG) criteria. While in REM sleep the EEG is characterized by high frequency, low amplitude activity; NREM sleep, by contrast, has an abundance of low frequency, high amplitude oscillations. These rhythmic activities are oscillations of varying frequency, amplitude and duration, including “slow oscillations,” “slow waves,” and “sleep spindles” (see Box 1 for a description of the neuronal circuits thought to generate these activity patterns).

Box 1 **Neural circuits underlying sleep dynamics.**

The mammalian NREM sleep EEG is characterized by the expression of slow waves that include the cortical slow oscillations (<1 Hz) and delta oscillations (1–4 Hz) (Buzsáki, 2006). While earlier reports have proposed that these two dynamics are distinct, recent experiments have suggested that they may be expression of the same cellular process: cortical neuronal UP and DOWN states (Timofeev and Chauvette, 2011). During slow oscillations the membrane potential of neocortical neurons is characterized by rhythmic fluctuations between two main states, the UP and the DOWN states at frequency <1 Hz (Contreras and Steriade, 1995). While DOWN-state transitions are characterized by the absence of synaptic inputs and membrane potential stability close to a value corresponding to the membrane resting potential, the UP state is a 0.200–1.5 s-long 15–20 mV depolarization during which action potential firing occurs. UP states are generated by bursts of synaptic inputs and are blocked by the voltage-gated sodium channel blocker tetrodotoxin (TTX). Moreover, UP- and DOWN-state transitions can be generated in cortical slices (Sanchez-Vives and McCormick, 2000) or surgically isolated cortical slabs *in vivo* (Timofeev et al., 2000). Although these initial observations suggested that cortical circuits are sufficient to generate this type of network oscillation, it must be considered that afferent fibers from other brain regions modulate UP- and DOWN-state generation in the cortex. In fact, incoming sensory inputs from the thalamus have been shown to trigger cortical UP-state transitions *in vivo* (Steriade et al., 1993) while electrical stimulation of thalamocortical axons reliably generates cortical UP-states in thalamocortical slice preparation (MacLean et al., 2005). Further, while much of the earlier work characterizing these cortical states had been performed under anesthesia, recent work in the head-fixed preparation has corroborated the cellular basis of the cortical slow oscillation (Chauvette et al., 2011).

In addition to slow waves, sleep spindles are characteristic features that are present in NREM sleep. These phasic 11–15 Hz oscillations, of approximately 0.5–3 s duration, appear in the EEG at a frequency of 0.1–0.2 Hz (Steriade, 2000). Spindles are thought to be generated by the thalamic reticular nucleus (TRN), a shell of GABAergic neurons that surround the dorsal thalamus and exert powerful synaptic inhibition on thalamocortical relay neurons (Bazhenov et al., 2000; Halassa et al., 2011). TRN and relay neurons express T-type Ca^2+^ channels, allowing them two modes of firing: tonic firing and bursting. At a depolarized membrane potential above the resting potential of −65 mV, these cells fire single Na^+^ spikes (tonic firing). But when hyperpolarized below the resting potential, they exhibit T-type Ca^2+^ channel-mediated spikes each characterized by a large Ca^2+^ spike crowned by high frequency bursts of Na^+^ spikes at a frequency of ~300 Hz (McCormick and Bal, 1997). It is thought that rhythmic bursting of the TRN at the spindle frequency entrains the thalamus by rhythmically de-inactivating T-type Ca^2+^ channels, causing thalamic relay neural populations to oscillate at the spindle frequency and thereby drive the neocortex (Timofeev and Chauvette, 2011). Recent evidence also underlies the importance of the corticothalamic interactions in the regulation of spindles (Bonjean et al., 2011).

Among the many functions attributed to sleep, one of the key ones is its role in memory. Two main hypotheses have been proposed to explain the finding that sleep appears to enhance memory. The *synaptic scaling hypothesis* suggests that the neocortex undergoes net synaptic potentiation during waking experiences, due to sensory-driven synaptic activity and increased release of neuromodulators (Tononi and Cirelli, 2003). According to this hypothesis, sleep evolved as a mechanism to downscale synapses while preserving their relative weights, balancing energy and space demands of the brain. This model is supported by several biochemical and electrophysiological observations. For example, recordings from layer II/III neocortical pyramidal neurons have shown that both the amplitude and frequency of miniature excitatory postsynaptic currents (mEPSCs) increase as a function of wakefulness, and decrease as a function of sleep (Liu et al., 2010). Phosphorylation at Ser831 of the AMPA glutamate receptor 1 subunit (GluR1) in cortical synaptoneurosomes—a change that increases single AMPA channel conductance and is known to be induced by synaptic potentiation—is enhanced by wakefulness, and reversed by sleep (Vyazovskiy et al., 2008). *In vivo* recordings of the neocortex in the freely behaving preparation have shown that neural firing rates (Vyazovskiy et al., 2009) and trans-callosal synaptic transmission (Vyazovskiy et al., 2008) exhibit a similar bidirectional modulation by wake and sleep.

By contrast to the synaptic scaling hypothesis, the *memory trace reactivation hypothesis* suggests that sleep is a state in which memories are consolidated by offline reactivation of the neurons involved in memory encoding during recent wakefulness (Lee and Wilson, 2002). This hypothesis is supported by recordings of neuronal ensembles from the hippocampus, where sequences of pyramidal neuronal firing encoding spatial trajectories during active behavior are replayed during subsequent sleep (Lee and Wilson, 2002). Replay events occur during 200–300 Hz oscillations of the hippocampal local field potential (LFP) known as ripples, whose electrical disruption has been shown to attenuate spatial memory (Girardeau et al., 2009).

Recent observations that astrocytes actively modulate brain rhythms thought to mediate these two processes, raise the need for computational studies of astrocytic function in order to fully understand the outcome of these two possible sleep functions.

## Local expression of sleep dynamics

While EEG tends to provide excellent information about the precise timing of brain events (i.e., excellent temporal resolution), the location in the brain where these events are taking place are relatively poor (i.e., limited spatial resolution). However, the development of high-resolution monitoring of neural activity had major impact on the study of sleep. High-density EEG recordings have shown that slow oscillations occurring during NREM sleep are traveling wave originating from frontal cortical areas (Massimini et al., 2004). Intracranial recordings in patients undergoing epilepsy surgery demonstrated that both slow waves and sleep spindles are expressed in a spatially confined manner, rather than simultaneously occurring across multiple cortical regions (Nir et al., 2011). Asynchronous spindle generation has also been corroborated by magnetoencephalography (MEG), revealing distinct spindles that may be obscured in EEG recordings (Dehghani et al., 2010). Moreover, several studies have shown the occurrence of sleep dynamics in localized brain regions during waking behavior (Vyazovskiy et al., 2011) and the occurrence of waking dynamics in localized brain areas during sleep (Nobili et al., 2011). These discoveries paint a new picture of brain state regulation adding further complexity than previously thought (Rector et al., 2009; Krueger and Tononi, 2011). Sleep may be better described as a continuous variable along a state-spectrum. In addition, graded activation of state-controlling microcircuits may be utilized to achieve diverse outcomes pertinent to processes such as working memory and selective attention (Harris and Thiele, 2011).

An intriguing question is the mechanism by which this local regulation of brain dynamics occurs. It is known that local increases in slow waves are dependent on prior behavior (Huber et al., 2004, 2006). For example, training on a motor task that requires hand movement has been shown to result in disproportionate increase of slow waves in the contralateral sensory-motor cortex (Huber et al., 2004). This suggests that the expression of slow waves could either be driven by local circuitry, or be modified by it. Further, the amplitude of slow waves might be dependent on the underlying local synaptic weights (Esser et al., 2007). Therefore, local modulators of synaptic transmission could serve as ideal candidates for mediating local generation of sleep dynamics. Below, we discuss anatomical and functional evidence suggesting that astrocytes are ideal candidates for providing local modulation of sleep dynamics. Among the different functions that astrocytes play in the brain, we will focus our attention on the process of neuroactive molecule release.

## Astrocyte regulation of synapses

In the last 20 years, astrocytes have been shown to directly release chemical transmitters in a process termed “gliotransmission” (Zhang and Haydon, 2005). In this process, astrocytes release neurotransmitters, cytokines, peptides, and neuromodulators that can provide an important regulatory feedback to neurons (Halassa and Haydon, 2010).

Astrocytes express the enzyme serine racemase (Wolosker et al., 1999a,b), a protein that converts the amino acid L-serine to D-serine. D-serine is known to bind the glycine-site of the N-methyl-D-Aspartate receptor (NMDAR) with four times the affinity of glycine (Wolosker et al., 1999a,b) (Figure 2C). The impact of astrocytic D-serine on synaptic transmission and plasticity has been mostly studied in the hypothalamus and the hippocampus, where the proximity of astrocytic processes to neurons determines D-serine availability to the synapse, and thus the ability of the synapse to express NMDAR-dependent plasticity (Panatier et al., 2006; Henneberger et al., 2010). More recently, studies in the mouse neocortex *in vivo* have shown that astrocytic Ca^2+^ signaling and associated D-serine release are important for translating cholinergic modulatory signals to NMDAR-dependent cortical plasticity (Takata et al., 2011).

ATP released from astrocytes can modulate neurons directly through the activation of P_2_ receptors (Gordon et al., 2009; Gourine et al., 2010). Alternatively, after rapid degradation to adenosine by ectonucleotidases (Dunwiddie et al., 1997; Dunwiddie and Masino, 2001), it can modulate synaptic transmission through the activation of adenosine receptors (Pascual et al., 2005; Serrano et al., 2006; Panatier et al., 2011). Astrocytic ATP can be stored in secretory compartments that fuse in a SNARE-dependent fashion upon an increase of the intracellular Ca^2+^ concentration (Coco et al., 2003; Zhang et al., 2007). Using a mouse model in which SNARE-dependent gliotransmission was impaired (dnSNARE mouse), Pascual et al. showed that astrocytes release ATP to control the strength of hippocampal synapses through its metabolite adenosine (Pascual et al., 2005) (Figure 2B). Using extracellular field recordings and brain slice preparation, these authors found that basal synaptic transmission was increased in transgenic mice, compared to wild type (WT) mice, a phenotype that was mimicked by the application of the A_1_-receptor antagonist, DPCPX (or CPT) in WT animals. In contrast, application of the A_1_ receptor agonist, CCPA, partially recovered the phenotype in transgenic mice, causing the reduction of fEPSP. Evidence that adenosine is generated by degradation of ATP comes from the observation that in slices from WT animals, the application of the ectonucleotidase inhibitor ARL67156 caused an inhibition of synaptic transmission that was blocked by the P_2_ receptor antagonist RB-2. This effect was absent in transgenic mice. Moreover, using a luciferine-luciferase assay, extracellular ATP levels were found to be reduced in transgenic mice, compared to controls.

The same study (Pascual et al., 2005) provided evidence that astrocytes release ATP in two modes: *tonic* release, which leads to a persistent synaptic suppression, and *phasic* release, which modulates synaptic plasticity when activity-dependent recruitment of astrocytes occurs. More specifically, it was demonstrated that glia-derived adenosine is responsible for activity-dependent heterosynaptic depression at excitatory synapses through A_1_ receptors (Pascual et al., 2005). Thus, astrocytes operate as sensors of neuronal activity and provide an activity-dependent phasic and tonic modulatory feedback to synapses at a local scale. On a longer timescale, adenosine acting through adenosine A_1_ receptors has been shown to control the surface expression of postsynaptic NMDA receptors (Deng et al., 2011), suggesting that astrocytic modulation of synaptic physiology can span multiple timescales. How this modulation relates to neural network function and behavior has just become possible to study (Fellin et al., 2009; Halassa et al., 2009), given the advances in molecular genetics and astrocyte-specific manipulations. Below, we review the first few studies that have started addressing this topic.

## Astrocytic regulation of neuronal circuits and sleep

**Figure 3:**
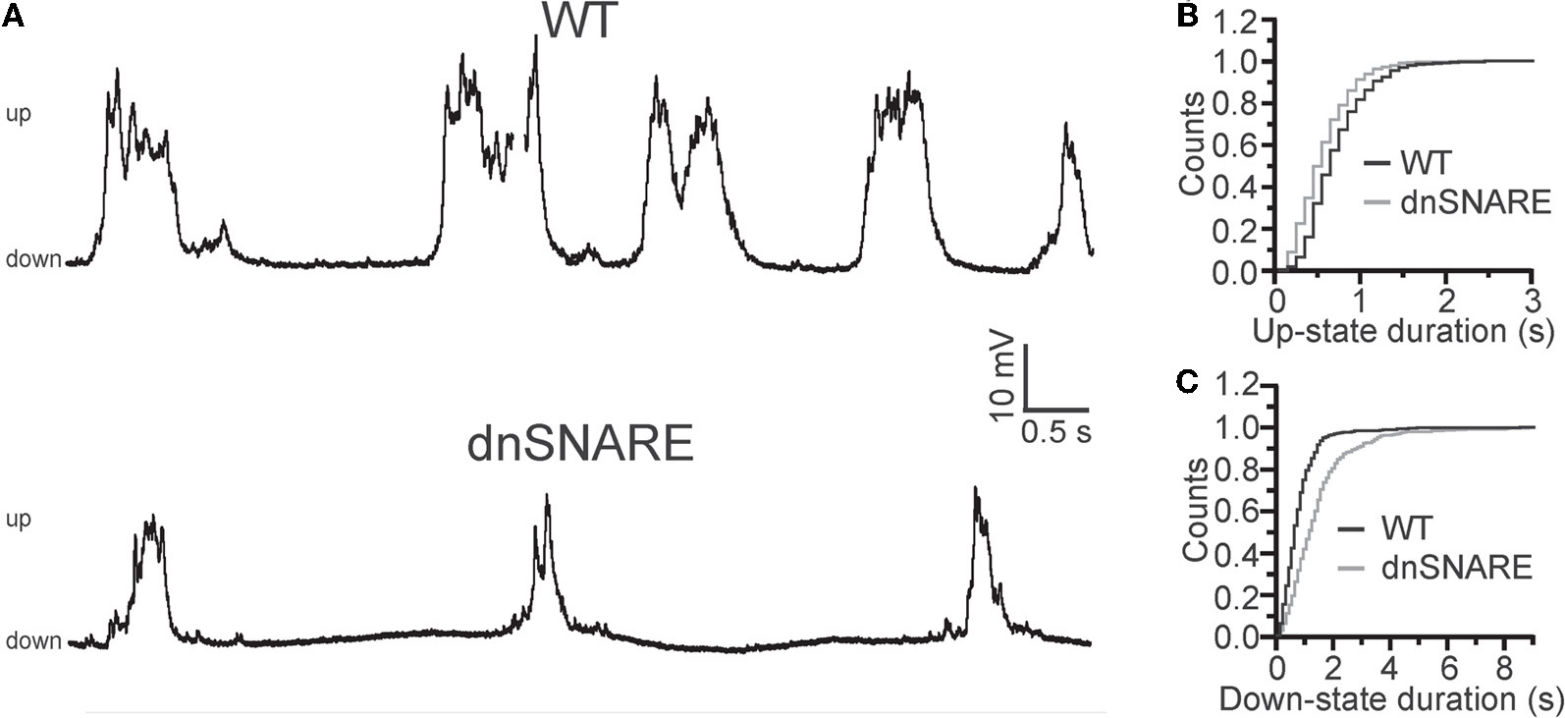
**Astrocytes modulate slow oscillations. (A)** Representative patch-clamp recordings from WT (*top*) and dnSNARE (*bottom*) anesthetized animals *in vivo*, showing reduced slow oscillations in transgenic mice with impaired gliotransmission (dnSNARE). **(B,C)** Cumulative distribution showing shorter UP **(B)** and longer DOWN- **(C)** state durations in dnSNARE mice (gray line) compared to WT controls (black line). Modifed from Fellin et al. (2009).

The first study to demonstrate an impact of astrocytes and gliotransmission on brain dynamics used the dnSNARE mouse (discussed above) showing that impairment of vesicular gliotransmission attenuates cortical slow oscillations (Fellin et al., 2009). Intracellular patch-clamp recordings *in vivo* revealed that the reduction of the slow oscillations was due to decreased UP-state probability of cortical neurons. (See Box 1 for definition of UP and DOWN states). UP-state transitions were found to be shorter and DOWN-state transitions longer in transgenic mice compared to controls (Figure 3), leading also to a reduction in the frequency of UP-state transitions. In contrast, the maximal amplitude of the UP-state was unaffected by transgene expression. Importantly, the reduction in slow oscillations was confirmed by chronic EEG recordings in freely behaving mice during natural sleep (Fellin et al., 2009). Following sleep deprivation, the power of slow oscillations in NREM sleep was selectively reduced in these animals. These network effects were the consequence of astrocytic modulation of intracortical synaptic transmission at two sites: a hypofunction of postsynaptic NMDA receptors, and by reducing extracellular adenosine, a loss of tonic A_1_ receptor-mediated inhibition. Indeed, AMPA/NMDA current ratio was found to be increased in transgenic mice compared to controls. This was due to selective decrease in NMDA receptor current due to impaired D-serine release and reduction in the surface expression of NMDA receptors. Moreover, application of the adenosine receptor antagonist CPT caused a significant increase in synaptic transmission in slices derived from WT—but not transgenic animals—demonstrating that the tonic level of adenosine was dependent on astrocytic gliotransmission. This dual astrocytic regulation at synaptic level correlated with the observed changes in network activity recorded in living animals. *In vivo* application of CPT caused an increase in slow oscillations in WT animals, but not in transgenic animals, while application of the NMDA receptor antagonist D-AP5 caused a significant decrease in slow oscillation power in WT but not in transgenic mice. Based on these results, it was concluded that astrocytic regulation at the synaptic level translates into active feedback at the neuronal-network level (Fellin et al., 2009). It is important to note that astrocytic regulation of network UP-states has also been confirmed by a recent *in situ* study (Poskanzer and Yuste, 2011), and that a role of adenosine in the regulation of low frequency rhythmogenesis has been recently described in the thalamus (Lorincz et al., 2009). In this latter study, infra-slow oscillations were observed to be strongly regulated by ATP and by its metabolite adenosine. Although not tested directly in that study, thalamic astrocytes could be a potential cellular source of this nucleoside.

To address the impact of slow wave reduction on sleep behavior, chronic polysomnography was implemented in the dnSNARE mice and their WT littermates. The major finding was that slow-wave homeostasis was reduced in the dnSNARE mice under baseline conditions and was accentuated after sleep deprivation (Halassa et al., 2009). This electrophysiological phenotype was accompanied by altered sleep homeostasis, where dnSNARE mice failed to exhibit an increased in sleep time following 6 h of sleep deprivation. Consistent with other studies (Pascual et al., 2005; Fellin et al., 2009), the effect of transgene expression was mimicked by intraperitoneal injection of CPT in WT animals. Moreover, attenuation of gliotransmission resulted in the alterations of the cognitive impairments following sleep deprivation assessed with a novel object recognition test, an effect that was phenocopied in WT animals by injection of CPT.

Taken together, these data show that astrocytic purines regulate synaptic transmission. By doing so, they also regulate network dynamics, cortical low-frequency rhythmogenesis and sleep itself. As described previously, although astrocytes are organized in a cellular syncytium, single cells occupy separate domains and lack long-range cellular projections. Thus, by releasing chemical transmitters, astrocytes may function as local modulators of neurons and networks and these processes might have important implication in sleep. For example, local increase in extracellular adenosine following ATP release in the thalamus and cortex may profoundly impact the generation of thalamocortical dynamics. Although adenosine generated by astrocyte-released ATP was shown to act mainly on synaptic transmission (but see Panatier et al., 2011), astrocyte-derived adenosine could also directly impact the intrinsic properties of thalamic cells by activating somatic A_1_ receptors leading to cell hyperpolarization. The ability to manipulate neuronal firing and intracellular signaling of neurons and astrocytes by optogenetic technologies (Boyden et al., 2005) will undoubtedly open the door to answering these and other questions on the role of neuron-glial control of brain activity.

## Astrocytic modulation of networks: implication for modeling studies

The experimental findings discussed above open new venues in computational modeling of network activity, and suggest that obtaining an accurate description of synaptically connected networks requires the inclusion of local astrocytic neuromodulation. In particular, previous computational studies showed that the strength of synaptic connections is crucial to regulate network synchronization during slow oscillation activity (Bazhenov et al., 2002; Compte et al., 2003; Esser et al., 2007). This is well predicted by the theory that synchronization of coupled oscillators is heavily influenced by the strength of the coupling (Pikovsky et al., 2001; Wang, 2010). Therefore, given the importance of the astrocyte in regulating synaptic physiology, it is obvious that realistic modeling of slow oscillation activity needs to include astrocytic neuromodulation.

In the remainder of this review, we will focus on two aspects of gliotransmission that we believe critical for future development of modeling studies: temporal and spatial complexity of astrocytic neuromodulatory feedback to networks. While the question of how to define connectivity in neuron-glia networks remains open, arguably the path to the proper answer rests in the distinction and appreciation of the different temporal and spatial scales underlying astrocytic neuromodulation. Precise understanding of these two aspects of astrocyte-to-neuron communication will be essential for the establishment of realistic and constrained models.

## Temporal aspects of gliotransmission

Evidence that gliotransmission operates on different time scales adds levels of complexity to our understanding of brain networks, and provides a challenging framework for experimentation and theory alike. For example, phasic ATP release from astrocytes provides an adenosinergic, activity-dependent heterosynaptic depression. Concurrently, tonic astrocytic adenosine mediates a constant suppression of presynaptic terminals through the activation of A_1_ receptors (Pascual et al., 2005; Halassa et al., 2009). Thus, the time scales of activity of gliotransmitters may be different and the phasic or tonic action must be included in a model as a crucial parameter.

**Figure 4:**
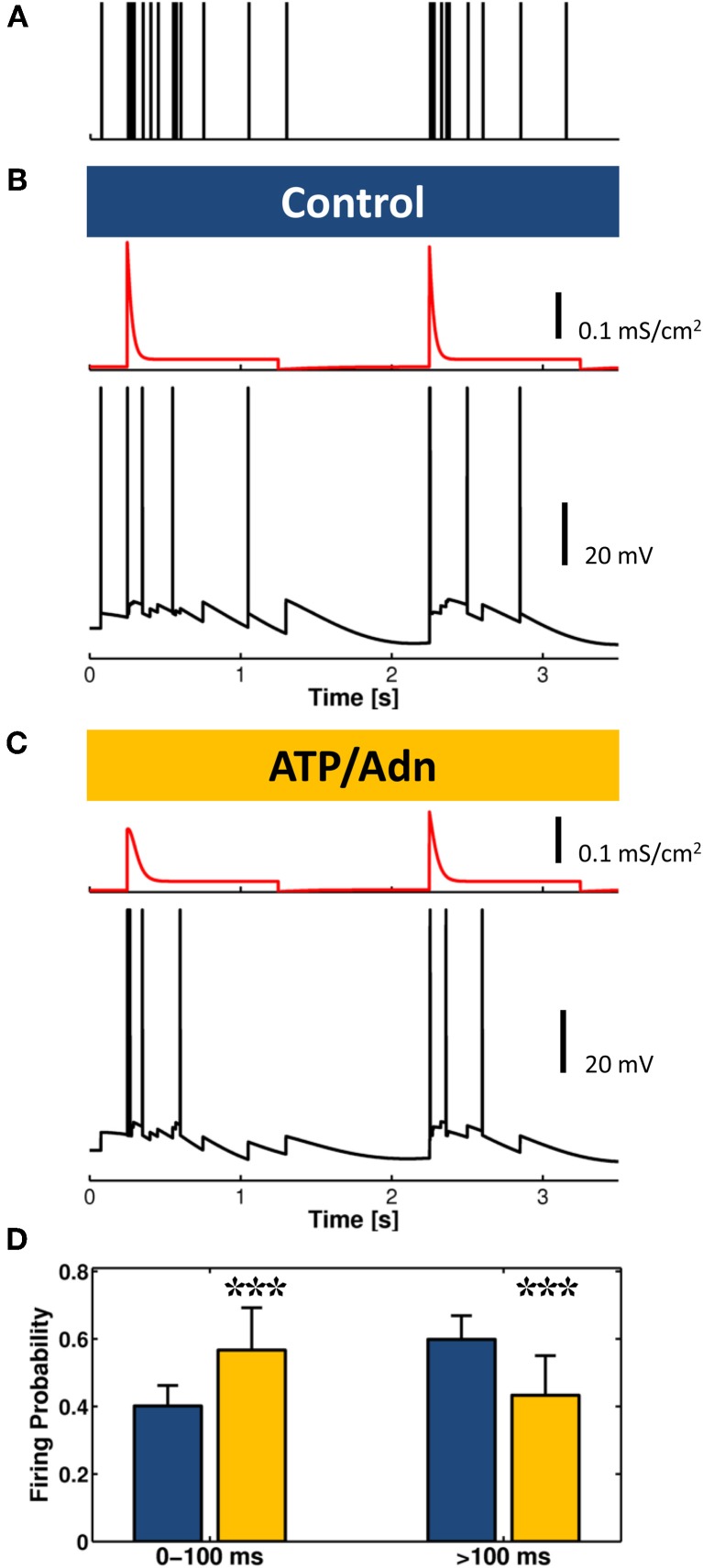
**Weakening of synaptic transmission by astrocyte-derived ATP and adenosine could promote bursting at the onset of the UP state. (A)** A stereotypical synaptic input alternating a phase of intense presynaptic firing to a relative quiescent phase, reminiscent of UP and DOWN states, respectively, is fed into a model of cortical neuron. **(B)** In control conditions, for low extracellular levels of ATP/adenosine (Adn), synaptic release probability is high, and the average synaptic conductance (*red traces*) is shaped by short-term depression while neuron fires (*black trace*) at sustained rate during the UP state. **(C)** For increased levels of ATP/Adn, the firing rate dramatically decreases due to the upstream reduction of these purines of synaptic release probability, but as shown in the histogram in **(D)**, due to the modulation of the synaptic filtering characteristics by increased extracellular ATP/Adn concentration, the neuron fires a burst of actions potentials at the onset of UP states at higher frequency than in **(B)** in control conditions (*n* = 100; Bar + Error bar: Mean + STD; χ^2^ test, *p* < 0.001). Synaptic release and ATP/Adn modulation of it were modeled as in panel **4A** in De Pittà et al. (2011). Postsynaptic currents were computed as the product of postsynaptic conductance and membrane voltage. Each input spike contributed to a change of postsynaptic conductance proportional to the amount of synaptically-released resources by a α-function such as α (*t*) = g_max_ · exp(1 − *t*/τ) · *t*/τ (Ermentrout and Terman, 2010) with *g*_max_ = 500 mS/cm^2^ and τ = 20 ms. The postsynaptic neuron was modeled as a regular spiking (RS) neuron according to (Destexhe, 2009).

In the context of slow oscillations, phasic release of ATP and D-serine by astrocytes (Panatier et al., 2006; Zhang et al., 2007) may modulate cortical UP and DOWN states. In the model originally developed by Hill and Tononi (2005), the depolarizing influence of NMDA currents plays an important role in maintaining the UP state as well as its synchrony among different neurons. This is likely due to the broadening of dendritic integration time constants by NMDA currents (Gasparini and Magee, 2006). The slow decay of NMDA currents could indeed account for integration of the highly heterogeneous synaptic inputs observed during UP states (McCormick et al., 2003), providing sufficient tonic drive to maintain neuronal firing persistence (Wang, 1999). Thus, a transient increase of NMDA currents due to the phasic release of D-serine from the astrocyte could promote neuronal firing, favoring synchrony and prolonging the duration of the UP state. On the other hand, this effect could be contrasted by transient weakening of synaptic transmission by phasic release of ATP and the subsequent accumulation of adenosine (Pascual et al., 2005; Serrano et al., 2006; Panatier et al., 2011). Another intriguing possibility is that astrocytic adenosine modifies synaptic filtering properties affecting the switch between short-term depression or facilitation (Abbott and Regehr, 2004). This scenario was recently addressed in a theoretical study by De Pittà et al. (2011): astrocytic control of the mode of synaptic plasticity was shown to depend on astrocyte-mediated modulation of synaptic filtering. One prediction of this study is that astrocyte-derived adenosine could favor the induction of short-term synaptic facilitation in response to incoming patterned activity, in agreement with experimental results (Pascual et al., 2005). In Figure 4, we modeled the firing activity of a cortical neuron triggered by a sample train of synaptic inputs reminiscent of UP and DOWN states (Hill and Tononi, 2005; Destexhe, 2009) under low (Control) and high (ATP/Adn) concentration of extracellular purines, which mimics increased extracellular adenosine concentration mediated by astrocytes during wakefulness (Schmitt et al., 2012). In the latter case, neuronal firing is reduced because of the upstream decrease of synaptic release probability by the purines which results in a weakened averaged synaptic conductance (*red traces*). However, under these same circumstances, the neuron mostly fires at the transitions from DOWN to UP states and the firing rate is higher at these transitions due to the modified synaptic filtering characteristics. On a network level, this process may promote synchrony, providing a potential local mechanism to trigger UP states at every cycle of the slow oscillations (Crunelli and Hughes, 2009). *In vitro*, for example, the ensuing inhibiting action of gliotransmission on synaptic release could account for the occurrence of synchronized bursting events and the observed neuronal firing statistics (Volman et al., 2007).

The interplay of tonic vs. phasic and activity-dependent regulation of synaptic transmission by the number of gliotransmitters that can be released from astrocytes is an important aspect to consider when studying how astrocytes shape neuronal network activity (Volman et al., 2007; Nadkarni et al., 2008; Silchenko and Tass, 2008; De Pittà et al., 2011). It is intriguing to consider how a single gliotransmitter can have contrasting effects on synaptic transmission. On one hand, astrocytic adenosine reduces synaptic transmission by binding to presynaptic A_1_ receptors (Pascual et al., 2005). On the other hand, activation of A_1_ receptors increases surface expression of postsynaptic NMDARs and synaptic strength (Deng et al., 2011). What is the computational value of a tonic reduction of presynaptic release probability while slowly increasing postsynaptic NMDA receptor currents? An intriguing possibility could be *synaptic redistribution* (Markram and Tsodyks, 1996). According to the synaptic-scaling hypothesis of sleep (Tononi and Cirelli, 2006), plastic processes occurring during wakefulness result in a net increase of synaptic strength in forebrain circuits. At individual synapses, such potentiation is likely regulated by spike-timing dependent plasticity (STDP). But at the network level, additional mechanisms might be required to dynamically regulate the progressive increase of synaptic strength and counteract its tendency to destabilize postsynaptic firing rates, either reducing them to zero or increasing them excessively (Abbott and Nelson, 2000). Redistribution of synaptic weights by presynaptic reduction of release probability in parallel to postsynaptic increase of NMDA receptors could be essential. Through this mechanism, synaptic potentiation could occur without increasing the firing rates of postsynaptic neurons or the steady-state excitability of recurrent networks, and at the same time optimizing STDP-mediated potentiation (Abbott and Nelson, 2000). Moreover this mechanism would allow to prevent energy consumption by otherwise undesired network firing (Buzsáki et al., 2002; Tononi and Cirelli, 2006). As such, synaptic redistribution could play a role during wakefulness similar to that hypothesized for synaptic scaling during sleep (Tononi and Cirelli, 2006). On the other hand, it is likely that the two processes are linked. Although the underlying signaling pathways remain to be elucidated, this possibility is corroborated by the recognition of a role of astrocytes in homeostatic brain functions mediated by TNFα (Turrigiano, 2006). TNFα released from astrocytes (Bezzi et al., 2001; Beattie et al., 2002) has been implicated in synaptic scaling (Beattie et al., 2002; Stellwagen and Malenka, 2006). Moreover, TNFα has also been reported to control gliotransmission at granule cell synapses in the dentate gyrus (Santello et al., 2011). Computational models that include regulation of ambient TNFα by astrocytes could provide predictions on the role of gliotransmission during the whole sleep cycle (Krueger, 2008).

## Spatial aspects of gliotransmission

Astrocytes are functionally organized cells, and different subcellular regions of astrocytic processes could locally provide different modulatory feedback on neighboring synapses (Fellin et al., 2004; Volterra and Meldolesi, 2005; Marchaland et al., 2008; Di Castro et al., 2011; Panatier et al., 2011). From a modeling viewpoint, this scenario subtends the notion that the “synaptic island” represented by a single astrocyte is intrinsically non-linear in its function and modulates neuronal networks on different time scales and also on distinct spatial scales (Goldberg et al., 2010; Bernardinelli et al., 2011).

Transient Ca^2+^ elevations at astrocytic processes can occur either spontaneously (Hirase et al., 2004; Sasaki et al., 2011) or be triggered by local synaptic activity (Wang et al., 2006), and in turn can locally release ATP (Di Castro et al., 2011; Panatier et al., 2011). Under proper conditions, however, Ca^2+^ signals may propagate along astrocytic processes to other processes of the cell and eventually to the whole cell, thus potentially allowing purinergic gliotransmission to occur on a much wider spatial scale (Panatier et al., 2006). Moreover, Ca^2+^ signals can propagate to other neighboring astrocytes triggering ATP release from other astrocytic cells (Volterra and Meldolesi, 2005). Despite numerous modeling studies put forth to account for the rich spatial dynamics of astrocyte Ca^2+^ signaling (Bennett et al., 2008; De Pittà et al., 2009; Goldberg et al., 2010; Dupont et al., 2011), a comprehensive theoretical framework that attempts to link local, functionally organized Ca^2+^ signals to global, whole-cell Ca^2+^ signals and intercellular Ca^2+^ propagation is still missing. Also, the origin of Ca^2+^ signals (i.e., intracellular vs. extracellular) and how it links to different types of gliotransmission remains to be fully elucidated.

## Conclusions

While much of the research on astrocyte-neuron interactions in the last 15 years has focused on the regulation of synaptic transmission by the astrocyte, recent experiments show that these cells are essential modulators of network activity. Initial reports have shown astrocytic modulation of the cortical slow oscillation, a fundamental brain dynamic observed in sleep and thought to be important for sleep's functions. The involvement of astrocytic modulation in sleep circuits makes mechanistic sense: astrocytes are slower signaling cells compared to neurons and may mediate activity-dependent changes of network function over extended periods of time. These discoveries come at an exciting time in astrocyte research, where our understanding of astrocytes function in the brain is expanding exponentially. Moreover, these new findings call for new and more elaborate *in silico* models of astrocyte-to-neuron communication that will move from the astrocytic modulation of single synapses to that of networks of synaptically connected neurons. In addition to providing fundamental insight into how the brain operates, further development of the experimental and theoretical knowledge of astrocyte role in the regulation of neuronal circuits will be of tremendous translational impact.

### Conflict of interest statement

The authors declare that the research was conducted in the absence of any commercial or financial relationships that could be construed as a potential conflict of interest.
